# Phycodris Fimbriata Extract Attenuates UVA-Induced Photoaging in Dermal Fibroblasts by Modulating Antioxidant Enzymes and ERK1/2 Pathway

**DOI:** 10.3390/cimb48100971

**Published:** 2026-09-22

**Authors:** Joonghyun Shim

**Affiliations:** Department of Biohealth-Convergence, Seoul Women’s University, Nowon-Gu, Seoul 01797, Republic of Korea; shimjh@swu.ac.kr

**Keywords:** *Phycodris fimbriata*, ultraviolet, collagen, MMP1, anti-aging

## Abstract

This study investigated the photoprotective effects of the *Phycodris fimbriata* extract against ultraviolet A (UVA)-induced photoaging in normal human dermal fibroblasts (NHDFs). The effects of the extract on the mRNA expression of *COL1A1*, *COL3A1*, *MMP1*, *SODs*, *CAT*, and *GPXs*, as well as on the protein levels of type I collagen and MMP1, were evaluated. Real-time quantitative RT-PCR analysis revealed that *P. fimbriata* treatment upregulated the mRNA expression levels of *COL1A1*, *COL3A1*, *SOD2*, and *SOD3*. Furthermore, the extract effectively restored type I collagen protein levels in UVA-irradiated NHDFs while concomitantly attenuating both *MMP1* transcription and MMP1 protein secretion. Beyond extracellular matrix (ECM) remodeling, *P. fimbriata* exhibited potent free radical scavenging activity, effectively reducing intracellular H_2_O_2_ accumulation. *GPX1* and *GPX7* were identified as the primary antioxidant enzymes mediating this intracellular H_2_O_2_ clearance. UVA exposure drives cellular senescence via reactive oxygen species generation and the subsequent activation of ERK1/2 and NF-κB signaling pathways. Interestingly, *P. fimbriata* extract significantly suppressed the UVA-induced phosphorylation of ERK1/2, thereby rescuing COL1A1 expression and decreasing MMP1 expression. Collectively, these findings demonstrate that *P. fimbriata* extract exerts significant anti-aging and antioxidative effects in NHDFs.

## 1. Introduction

The skin is a protective organ that shields the human body from external factors by providing physical, chemical, and biological barrier functions. The skin is easily damaged because it is constantly exposed to various external factors [1,2]. Pollutants such as fine dust, microorganisms, and ultraviolet (UV) radiation are representative harmful factors. These factors generate reactive oxygen species (ROS) within cells, leading to cell damage or apoptosis and, furthermore, accelerating the aging process [1,2,3,4].

UV radiation, a major factor in skin photoaging, is divided into three categories based on wavelength: UVA (320–400 nm), UVB (290–320 nm), and UVC (200–290 nm) [1,4]. UVC is mostly absorbed by the ozone layer, while UVA and UVB reach the Earth’s surface and exert significant effects on the skin. UVB penetrates the epidermis and induces erythema in the skin. Compared to UVB, UVA has a longer wavelength and can penetrate more deeply into the dermis. Moreover, UVA increases ROS in dermal fibroblasts, decreasing type I collagen synthesis while increasing the production of matrix metalloproteinase-1 (MMP1), a collagen-degrading enzyme [2,5].

Several ingredients such as retinol, retinyl palmitate, and adenosine are widely recognized to improve skin elasticity and reduce the appearance of wrinkles via dermal fibroblast activation. Cosmetic products containing these ingredients have demonstrated clinical efficacy in improving wrinkles [6,7]. These ingredients can improve skin aging by promoting collagen synthesis or inhibiting MMP1 generation. Recently, extensive research has been conducted to identify anti-aging compounds derived from natural sources, such as marine algae. Marine algae possess unique organic molecules distinct from those found in terrestrial plants and exhibit antimicrobial and antihyperlipidemic effects. Additionally, they possess antioxidant, anticoagulant, and anticancer properties [8,9,10,11]. *Phycodris fimbriata* is a species of red algae belonging to Delesseriaceae. It is distributed along the coasts of Hokkaido in Japan, Sakhalin, the Kuril Islands, and the Pacific coasts of North America, and is also widespread along the coastal regions of Korea. The thallus of *P. fimbriata* is pinkish, its branches are irregularly divided in a pinnate pattern, and its leaf margins are lined with serrated projections. To our knowledge, to date, the biological activities of *P. fimbriata* have not been elucidated.

Marine bioactive substances include polysaccharides such as fucoidan and alginate, as well as peptides, pigments, and lipids such as eicosapentaenoic acid (EPA) and docosahexaenoic acid (DHA) [12]. Marine pigments like cartenoids and mycosporine-like amino acids exhibit cosmeceutical effects, such as anti-inflammatory, antioxidant, and photoprotective properties [13,14]. Moreover, bioactive peptides derived from marine sources are effective in inhibiting photoaging [15]. Bioactive substances extracted from marine organisms are drawing particular attention for their ability to alleviate oxidative stress—a major cause of skin aging—and inhibit inflammatory responses. These peptides neutralize ROS to exert photoprotective effects, thereby mitigating oxidative damage and subsequent collagen degradation and wrinkle formation [15]. However, whether *P. fimbriata* extract exerts anti-aging effects on human dermal fibroblasts remains unknown. Therefore, this study aims to investigate how *P. fimbriata* extract regulates the gene expression and protein production of *COL1A1*, *COL3A1*, and *MMP1*, which are associated with anti-aging. In addition, this study aims to determine whether *P. fimbriata* extract modulates intracellular antioxidant enzymes and to elucidate the signaling pathways involved in its potential anti-photoaging effects. Ultimately, this study explores the potential of *P. fimbriata* as a promising bioactive compound for future biomedical applications.

## 2. Materials and Methods

### 2.1. Experimental Materials and Cell Culture

The *P. fimbriata* extract was distributed by the Marine Bio Bank of the National Marine Biodiversity Institute of Korea (MABIK., Seochon, Republic of Korea; NP30180011, https://www.mbris.kr/biobank, accessed on 12 August 2026). The leaves of *P. fimbriata* were collected from the coastline of Goseong-gun, Gangwon-do, Republic of Korea, on 30 May 2017. This resource was collected via scuba diving; the latitude and longitude of the collection site are 38°27′ 36.9″ N, 128°28′ 03.0″ E. The collected samples were washed three times and then freeze-dried using an Operon freeze-dryer (FDT-8650, Operon, Gimpo-si, Republic of Korea). A mixture of 16.5 g of the dried sample and 500 mL of 70% ethanol was sonicated for 1 h at room temperature (RT) using a Cleaner Ultrasonic bath (WUC-N30H, DAIHAN, Wonju, Republic of Korea). The extract was then filtered through filter paper with a diameter of 320 mm (1202-320; Whatman, Sigma-Aldrich, Burlington, MA, USA). The filtrate was transferred to a volumetric flask and concentrated using a Buchi rotary evaporator R-300 system (CHNYO Scientific, Zhengzhou, China). The final amount of extract obtained from 16.5 g of dried plant material was 5.83 g, resulting in an extraction yield of 35.33%. The lyophilized extract was dissolved in a solution of ethanol:DMSO (1:1; *v*/*v*) to a concentration of 10 mg/mL for storage. For the experiments, the extract was diluted in DMEM.

Normal human dermal fibroblasts (NHDFs) were purchased from Cell Applications (San Diego, CA, USA). DMEM, fetal bovine serum (FBS), and antibiotics (penicillin/streptomycin) were purchased from Invitrogen (Carlsbad, CA, USA). NHDFs were maintained in DMEM supplemented with 10% FBS, 100 U/mL penicillin, and 100 μg/mL streptomycin. The NHDFs used in the experiments were within the fourth passage and were cultured at 37 °C with 5% CO_2_ in a fully humidified incubator (Thermo Fisher Scientific, Waltham, MA, USA).

### 2.2. DPPH Free Radical Scavenging Assay

The antioxidant capacity was evaluated by monitoring DPPH radical scavenging activity. A 0.1 mM DPPH working solution was prepared by dissolving the reagent in a mixture of methanol and distilled water (3:2, *v*/*v*; Sigma-Aldrich). Aliquots of *P. fimbriata* extract (50 μL) at various concentrations were introduced into 500 μL of the DPPH solution, and the mixture was incubated at room temperature for 20 min in the dark. The resulting absorbance was quantified at 450 nm utilizing an Epoch microplate spectrophotometer (BioTek, Santa Clara, CA, USA). L-ascorbic acid (Sigma-Aldrich, MA, USA) was employed as a reference standard. The percentage of radical scavenging effect was determined using the formula below:

Inhibition ratio (%) = [(absorbance of blank − absorbance of test sample)/absorbance of blank] × 100

### 2.3. UVA Irradiation

To induce photoaging, NHDFs were seeded into a 35 mm tissue culture dishes at a density of 1.5 × 10^5^ cells/well 24 h prior to UVA irradiation. After inoculating 1 mL of phenol-red-free DMEM (Welgene, Gyeongsan, Republic of Korea) into 35 mm tissue culture dish, the cells were exposed to UVA at a dose of 3 J/cm^2^ using a UVA irradiator (BLX-365; Vilber Lourmat, Collegien, France).

### 2.4. Cell Viability Assay

Cell viability was evaluated using the Cell Counting Kit-8 (CCK-8; Dojindo, Kumamoto, Japan). NHDFs (3 × 10^3^ cells/well) were seeded into 96-well cell culture plates and pre-incubated for 24 h. After a 24– or 48–h treatment with *P. fimbriata* extract in serum-free medium, CCK-8 reagent (diluted 1:10 in phenol red-free DMEM) was added to each well, followed by incubation for 1 h in a humidified incubator. The absorbance of the CCK-8 reagent was measured at 450 nm using a spectrophotometer (BioTek). The absorbance of wells containing only medium without cells was used as a blank.

### 2.5. Scratch Wound Healing Assay

NHDFs were plated in 35 mm culture dishes at a density of 1.5 × 10^5^ cells per dish and cultured to 80–90% confluence. The culture medium was then replaced with phenol red-free DMEM. The cells were exposed to UVA irradiation (3 J/cm^2^), followed by mechanical scratching using a 1 mL pipette tip (Thermo Fisher Scientific, MA, USA) to create a wound. Following two washes with phosphate-buffered saline (PBS; Welgene), the cells were incubated for 48 h in serum-free DMEM supplemented with *P. fimbriata* extract. To evaluate the wound-healing effect of the *P. fimbriata* extract, images of the same region under each condition were captured using a microscope (CKX53; Olympus, Tokyo, Japan) at 0, 24, and 48 h after treatment. In this experiment, the wound closure rate was normalized using the following formula.

% of Wound Closure = [(AT0 − ATx)/AT0] × 100

A: the area of the scratch wound; T0: is the initial time (0 h); and Tx: variable time point.

### 2.6. RNA Extraction and Real-Time Quantitative RT-PCR

Total RNA was extracted using TRIzol reagent (Thermo Fisher Scientific, MA, USA) according to the manufacturer’s protocol. The purity and integrity of the mRNA were assessed by measuring the A260/A280 ratio, and mRNA yield was determined by measuring absorbance at 260 nm using a spectrophotometer (BioTek). cDNA synthesis from the extracted RNA was performed using SuperiorScript III Master Mix (Enzynomix, Daejeon, Republic of Korea). To compare the relative expression levels of specific genes, real-time quantitative RT-PCR (RT-qPCR) was performed using the LightCycler^®^ 96 system (Roche, Basel, Switzerland). The TaqMan Gene Expression Assays (Thermo Fisher Scientific) utilized in this study are summarized in Table 1. For the amplification process, an initial denaturation step was performed at 50 °C for 120 s followed by 95 °C for 600 s. Subsequently, 45 cycles were executed, each comprising denaturation at 95 °C for 15 s and extension at 60 °C for 60 s. To determine relative gene expression levels, the 2^−ΔΔCt^ method was applied, with GAPDH serving as the internal reference for normalization.

### 2.7. Enzyme-Linked Immunosorbent Assay (ELISA)

To investigate the effect of *P. fimbriata* extract on the production of type I collagen and MMP1 proteins, 3 × 10^5^ fibroblasts were seeded in 60 mm cell culture dishes. After incubation for 24 h in a humidified incubator, the cells were exposed to UVA at a dose of 3 J/cm^2^ using a UVA irradiator. Following UVA irradiation, *P. fimbriata* extract diluted in DMEM was applied to the cells for 48 h. A procollagen type I c-peptide (PICP) ELISA kit (Takara Bio, Kyoto, Japan) and an MMP1 ELISA kit (Thermo Fisher Scientific) were used for this experiment. The amounts of type I collagen and MMP1 secreted into the conditioned medium were measured according to the manufacturers’ protocols.

### 2.8. H_2_O_2_ Production Measurements

Hydrogen peroxide (H_2_O_2_) released from UVA-irradiated NHDFs was quantified using the Amplex Red Hydrogen Peroxide/Peroxidase Assay Kit (Invitrogen), following the manufacturer’s protocol. NHDFs were pretreated with *P. fimbriata* extract for 24 h prior to UVA irradiation. Following UVA exposure, 50 μL of culture medium from each condition was collected and incubated for 10 min with 100 μL of a reaction solution containing 100 μM Amplex Red and 0.2 U/mL horseradish peroxidase. Fluorescence was measured using an Epoch microplate reader (BioTek) at an emission wavelength of 590 nm following excitation at 560 nm.

### 2.9. Protein Kinase Array Analysis

Phosphorylated protein profiles were analyzed using the Human Phospho-Kinase Array Kit (R&D Systems, Minneapolis, MN, USA), following the manufacturer’s instructions. Briefly, cell lysates (300 μg) were incubated with each array sheet overnight at 4 °C on a rocking shaker. The cell lysates were removed, and the array sheets were washed three times with washing buffer. The arrays were incubated with the primary antibody for 2 h at RT and washed again. The secondary antibody was then added and incubated for 1 h on an orbital shaker (Thermo Fisher Scientific, MA, USA). The arrays were washed a final time with washing buffer, and phosphorylated protein spots were detected using a chemiluminescent solution. The relative density of each phosphorylated protein spot was evaluated using the Multi Gauge V3.0 program (FujiFilm, Tokyo, Japan) by subtracting the background and the PBS-negative control values.

### 2.10. Western Blot

Cells were lysed on ice for 2 h with RIPA lysis buffer (Millipore, Burlington, MA, USA) containing a protease/phosphatase inhibitor cocktail (Merck, Massachusetts, MA, USA). Following incubation, cell lysates were centrifuged at 12,000 rpm for 15 min at 4 °C, and the supernatants were transferred to fresh microcentrifuge tubes. For immunoblot analysis, 20 μg of protein was separated using 4–12% Bis-Tris gradient gels and subsequently transferred onto a PVDF membrane (iBlot^TM^ 3 Transfer stack; Thermo Fisher Scientific). The membranes were blocked with Tris-buffered saline with Tween-20 (TBST) containing 1% bovine serum albumin (BSA; Sigma) for 1 h at RT. The PVDF membranes were incubated overnight at 4 °C with a GAPDH primary antibody (mouse monoclonal, 1:1000; Santa Cruz Biotechnology, Santa Cruz, CA, USA), an IκBα primary antibody (mouse monoclonal, 1:1000; Cell Signaling Technology, Boston, MA, USA) and a phosphorylated-IκBα primary antibody (mouse monoclonal, 1:1000; Cell Signaling Technology, USA) in TBST containing 1% BSA. The blots were washed with TBST and incubated with HRP-conjugated secondary antibodies (1:3000; Bio-Rad, Hercules, CA, USA) for 1.5 h at RT. The blots were developed using Clarity Western ECL blotting substrate (Bio-Rad), following the manufacturer’s instructions.

### 2.11. High-Resolution Liquid Chromatography–Mass Spectrometry (HR-LC/MS)

Twenty milligrams of freeze-dried *P. fimbriata* extract was added to 1 mL of extraction solvent (0.1% formic acid in 70% acetonitrile, Sigma-Aldrich, MA, USA), mixed for 30 s, and then sonicated for 30 min. The entire sample was filtered through a 0.45-μm syringe filter (PTFE) and centrifuged at 3000 rpm and 15 °C for 15 min; the resulting supernatant was used as the analytical sample. The analytical instruments used were the SII Xcalibur and Orbitrap Exploris 120 (Thermo Fisher Scientific), and the column used was the ACQUITY UPLC BEH C18 (Waters, Milford, MA, USA; 2.1 × 100 mm, 1.7 μm). The analysis was performed at a column temperature of 40 °C and a flow rate of 0.3 mL/min. Additional analytical conditions are specified in Table 2.

### 2.12. Statistical Analysis

Data were analyzed using appropriate statistical methods, and results are presented as the mean ± standard deviation (S.D.) of at least three independent experiments. Statistical significance among multiple groups was evaluated using one-way analysis of variance followed by Tukey’s post hoc test. A *p*-value of less than 0.05 was regarded as statistically significant. All statistical analyses were performed using SPSS Statistics version 29.0 (IBM Corporation, Armonk, NY, USA).

## 3. Results and Discussion

### 3.1. Analysis of NHDFs Viability After Treatment with Phycodris fimbriata Extract

To evaluate the cytotoxic concentration of *P. fimbriata* extract on NHDFs, a CCK-8 assay was performed. NHDFs were treated with *P. fimbriata* extract at concentrations of 1000, 100, 10, and 1 μg/mL, as well as 100, 10, and 1 ng/mL for 24 h (Figure 1A) and 48 h (Figure 1B). A significant decrease in NHDF viability was observed at concentrations higher than 100 μg/mL. Therefore, subsequent experiments were performed using *P. fimbriata* extract at concentrations of 10, 1, and 0.1 μg/mL.

### 3.2. Wound Healing Effect of P. fimbriata Extract

To investigate whether *P. fimbriata* affects the migration of NHDFs exposed to ultraviolet A (UVA) irradiation, a scratch wound-healing assay was performed. Compared with the control group, UVA-irradiated NHDFs showed a significant decrease in cell migration, indicating that UVA exposure negatively affects the migratory ability of NHDFs. In contrast, the *P. fimbriata*-treated group showed a significant increase in cell migration, effectively restoring the UVA-induced reduction in cell movement. This suggests that *P. fimbriata* extract has the potential to enhance NHDF migration and promote wound healing even after UVA-induced damage (Figure 1B). A scratch was made on the confluent cell monolayer, and migration was monitored for 48 h. Images of the same region under each condition were captured by a microscope at 0, 24, and 48 h after treatment. After 24 h of UVA exposure, NHDFs showed a 71% decrease in cell migration compared to the control group; however, in the group treated with *P. fimbriata*, cell migration increased by 2.68–fold times compared to the UVA-exposed group (Figure 1C). After 48 h of UVA exposure, NHDFs exhibited a 73% decrease in cell migration compared to the control group, whereas treatment with *P. fimbriata* extract increased cell migration 2.93 times compared to the UVA-exposed group, demonstrating that *P. fimbriata* extract significantly enhanced NHDF migration and promoted wound healing (Figure 1D).

### 3.3. Gene Expression Profile on NHDFs Following UVA Irradiation and Treatment of P. fimbriata Extract

After UVA irradiation, *P. fimbriata* extract was applied, and the expression patterns of the fibroblast marker genes *COL1A1*, *COL3A1*, and *MMP1* were analyzed by RT-qPCR. In the dermis, type I collagen accounts for approximately 80% and type III collagen approximately 15% of the total collagen content [16]. As aging progresses, the expression of collagen genes decreases, negatively affecting the elasticity of both the skin and muscles [17,18,19]. Upon UVA irradiation, NHDFs show a reduction in the expression of specific fibroblast markers, such as *COL1A1*, *COL3A1*, and *TIMP1*, compared to the untreated group, while *MMP1* expression is significantly increased [20,21]. Compared to the UVA-treated group, the group treated with 10 μg/mL of *P. fimbriata* extract showed a 1.84-fold increase in *COL1A1* expression and an approximately 4-fold increase in *COL3A1* expression (Figure 2A,B). The UVA-induced increase in *MMP1* expression was significantly reduced by 48%, 32%, and 39% following treatment with *P. fimbriata* extract at concentrations of 10, 1, and 0.1 μg/mL, respectively (Figure 2C).

### 3.4. Effects of P. fimbriata Extract on Type I Collagen Production and MMP1 Reduction

An enzyme-linked immunosorbent assay (ELISA) was used to analyze type I collagen and MMP1 protein levels in NHDFs (Figure 3). Type I collagen levels, which decreased by approximately 41% following UVA irradiation, increased by approximately 1.57-fold in the *P. fimbriata* extract-treated condition (Figure 3A). In addition, an MMP1-ELISA confirmed that *P. fimbriata* extract treatment significantly reduced MMP1 protein levels. MMP1 protein levels, which increased approximately 1.78-fold following UVA irradiation, decreased by approximately 34% upon treatment with *P. fimbriata* extract (Figure 3B). These results align with the RT-qPCR data regarding the expression of *COL1A1* and *MMP1* (Figure 2), indicating that *P. fimbriata* extract modulates both gene expression and protein production.

All-trans retinoic acid (ATRA), a widely recognized agent for treating skin conditions and mitigating photoaging, is known to promote collagen synthesis and suppress MMP1 expression [20,22]. Although ATRA effectively attenuates photoaging in three-dimensional (3D) models, such as dermal and skin equivalents [20], as well as in vivo in photoaged skin [22], its anti-photoaging effects are not significantly pronounced in 2D culture systems [21]. Given that the present study was designed to evaluate the photoprotective properties of *P. fimbriata* extract using a 2D culture approach, ATRA was excluded as a positive control. However, in this study, *P. fimbriata* extract regulated type I collagen and MMP1 levels in a manner similar to ATRA, suggesting the extract possesses anti-aging activity (Figure 2 and Figure 3).

### 3.5. Effect of P. fimbriata Extract on the Expression of Antioxidant Enzymes

Reactive oxygen species (ROS) generation is the initial signal that induces extracellular matrix-degrading enzymes, such as MMP1, in response to UV radiation [23]. To evaluate the antioxidant activity of *P. fimbriata*, a 2,2-diphenyl-1-picrylhydrazyl (DPPH) assay was performed. The DPPH assay confirmed that *P. fimbriata* extract exhibits antioxidant activity, with radical scavenging abilities of 40.3% and 22.5% at concentrations of 10 mg/mL and 1 mg/mL, respectively (Figure 4A). To further investigate whether *P. fimbriata* extract inhibits the production of hydrogen peroxide (H_2_O_2_), an Amplex Red assay was performed. The results indicated that UVA-irradiated NHDFs produced H_2_O_2_ levels approximately 3.9-fold higher than those of the control group, whereas *P. fimbriata* extract treatment significantly reduced H_2_O_2_ generation (Figure 4B).

Given that *P. fimbriata* extract attenuated UVA-induced H_2_O_2_ production, the intracellular antioxidant enzymes responsible for this reduction were investigated. Consistent with previous reports [21], the expression levels of *SOD2*, *SOD3*, and *CAT* genes were significantly decreased by UVA irradiation. Treatment with *P. fimbriata* extract (10, 1, and 0.1 μg/mL) resulted in 2.47-, 2.4-, and 2.49-fold increases in *SOD2* expression, respectively, compared with the UVA-treated group (Figure 5B). Furthermore, *SOD3* gene expression increased 1.93-fold in the presence of *P. fimbriata* extract compared with UVA treatment (Figure 5C).

Glutathione peroxidase (*GPX*) and *CAT* are responsible for reducing UVA-induced H_2_O_2_ levels. Since *CAT* gene expression was not restored by *P. fimbriata* extract treatment (Figure 5D), I examined the expression of *GPX*. The GPX family catalyzes the reduction of H_2_O_2_ to H_2_O [24,25]. The GPX family comprises eight members. GPX1 is highly expressed in fibroblasts, and fibroblasts from GPX1-knockout mice can undergo senescence-like H_2_O_2_-induced cell death. GPX7 possesses a unique structural feature in which cysteine replaces selenocysteine at the catalytic center in mouse embryonic fibroblasts [26]. GPX7-knockout mice exhibit a shortened lifespan, with oxidative DNA damage and apoptosis predominantly in the kidneys. In addition, multiple organ dysfunctions have been diagnosed, including cardiomegaly, splenomegaly, fatty liver, glomerulonephritis, and carcinogenesis [27,28]. Among the eight *GPX* subtypes analyzed using RT-qPCR, *GPX2*, *GPX5*, and *GPX6* expression was not detected in NHDFs. Interestingly, mRNA expression levels of *GPX1* and *GPX7* significantly increased in response to *P. fimbriata* extract treatment compared to UVA irradiation (Figure 6A,B), whereas *GPX3*, *GPX4*, and *GPX8* expression decreased with UVA irradiation and remained unaffected by *P. fimbriata* extract (Figure 6C–E). These results suggest that *P. fimbriata* extract may reduce intracellular H_2_O_2_ levels primarily through the modulation of *GPX1* and *GPX7*.

### 3.6. Analysis of Intracellular Signaling Pathways Modulated by P. fimbriata

UVA radiation induces intracellular ROS production and decreases the expression of antioxidant enzymes such as SOD, CAT, and GPX [29,30]. UV-induced ROS can induce skin aging through the activation of mitogen-activated protein kinases (MAPKs), including ERK, JNK, and p38, and the NF-kB pathway [31,32,33,34]. Activation of MAPKs leads to the phosphorylation of c-Jun, which subsequently activates the transcription factor AP-1, contributing to decreased type I collagen expression and increased MMP production [35,36]. I investigated the potential of *P. fimbriata* extract to inhibit UVA-induced activation of these MAPK and NF-κB pathways. UVA irradiation increased the phosphorylation of ERK1/2, p38, and IκBα. Notably, *P. fimbriata* extract treatment prevented the phosphorylation of ERK1/2 specifically, whereas it failed to inhibit the UVA-induced increase in p38 and IκBα phosphorylation (Figure 7). These results indicate that *P. fimbriata* extract modulates the signaling pathways involved in UVA-induced photodamage through the ERK1/2 pathway. To directly confirm that the recovery of COL1A1 expression and the increase in MMP1 expression—both of which were reduced by UVA treatment—are mediated by ERK1/2, it is necessary to modulate this signaling pathway using ERK1/2 inhibitors (e.g., U0126) or activators and to verify the expression levels of the various genes and proteins analyzed in this experiment [37].

### 3.7. Analysis of Unknown Compounds in P. fimbriata Extract Using HR-LC/MS

High-resolution liquid chromatography–mass spectrometry (HR-LC/MS) was employed to profile the chemical components of *P. fimbriata* extract. As shown in Figure 8, multiple peaks were observed in both positive and negative ionization modes. The unknown components were tentatively identified by comparison with the mzCloud (Compound Discoverer 3.5) and mzVault (mzVault 2.3 SP1) spectral libraries. In total, five compounds in positive ionization mode and three in negative ionization mode were selected based on a best-match score (BM) threshold of ≥80 against reference spectral libraries (Table 2).

In positive ionization mode, choline (a major cell membrane component and essential nutrient), betaine (a natural amino acid derivative functioning as a natural moisturizing factor [NMF]), proline (a key component of collagen), lyso-PAF-C16 (1-O-hexadecyl-2-hydroxy-sn-glycero-3-phosphocholine), and PC (16:0/0:0) (1-palmitoyl-2-hydroxy-sn-glycero-3-phosphocholine) were identified [38,39,40]. Choline, which can prevent angiotensin II-induced cardiac hypertrophy, inhibits ROS-mediated p38 MAPK activation in cardiac fibroblasts [40]. And betaine inhibits p-ERK activation in skeletal muscle cells [41]. The choline and betaine present in the *P. fimbriata* extract used in this study were found to inhibit p38 and p-ERK in fibroblasts and skeletal muscle cells, consistent with Figure 7A,C. This suggests that these two compounds play a crucial role in the extract, and further analysis is needed to elucidate their respective functions. In negative ionization mode, several compounds were identified, including 2-methylcitrate, which has been associated with skin metabolites in patients with psoriasis vulgaris, and 4-hydroxybenzoic acid, a common preservative in the cosmetic industry [42,43].

Nonetheless, these compound identifications should be considered preliminary, as they have not been quantitatively linked to biological activity. Future investigations are required to elucidate the specific constituents responsible for the anti-photoaging properties of *P. fimbriata* extract. Further validation studies utilizing quantitative LC-MS/MS and authentic reference standards are warranted in future investigations.

## 4. Conclusions

Extrinsic factors such as ultraviolet (UV) radiation and reactive oxygen species (ROS) chain reactions accelerate skin aging and contribute to the development of various skin disorders [4,5]. Furthermore, aging leads to a decline in the regenerative capacity and normal function of tissues due to a reduction in the number or dysfunction of resident cells in adult tissues [44,45].

This study determined the appropriate concentration of *P. fimbriata* extract for the treatment of human dermal fibroblasts and confirmed that concentrations under 10 μg/mL do not affect cell viability (Figure 1). *P. fimbriata* extract significantly restored the UVA-induced reduction in *COL1A1* and *COL3A1* gene expression and type I collagen protein production (Figure 2A,B and Figure 3A). Regarding MMP1 expression, *P. fimbriata* extract effectively reduced the UVA-induced upregulation of MMP1 at both the gene and protein levels (Figure 2C and Figure 3B).

In addition, *P. fimbriata* extract upregulated the expression of the antioxidant enzymes *SOD2*, *SOD3*, *GPX1*, and *GPX7*, suggesting they play a role in enhancing the cellular antioxidant defense system (Figure 5 and Figure 6). These intracellular enzymes contributed to the reduction in UVA-induced intracellular H_2_O_2_ levels (Figure 4B). ROS generated by UVA irradiation suppress *COL1A1* expression and induce *MMP1* expression via MAPK and NF-κB signaling [31,32,33,34]. *P. fimbriata* extract suppressed UVA-induced cellular aging by specifically inhibiting the p-ERK1/2 pathway within the MAPK signaling cascade (Figure 7).

These findings suggest that *P. fimbriata* extract has potential as a novel active ingredient for the mitigation of skin aging. A limitation of this paper is that the efficacy of the *P. fimbriata* extract was verified only through in vitro cell culture. Further studies using 3D artificial skin models and animal experiments are needed to confirm whether the results of this study can be replicated. Further in-depth studies are warranted to identify the various marker compounds present in *P. fimbriata* extract, determine which compounds contribute to its anti-aging effects on the skin, and elucidate the specific intracellular signaling pathways by which they mitigate UV-induced skin aging.

## Figures and Tables

**Figure 1:**
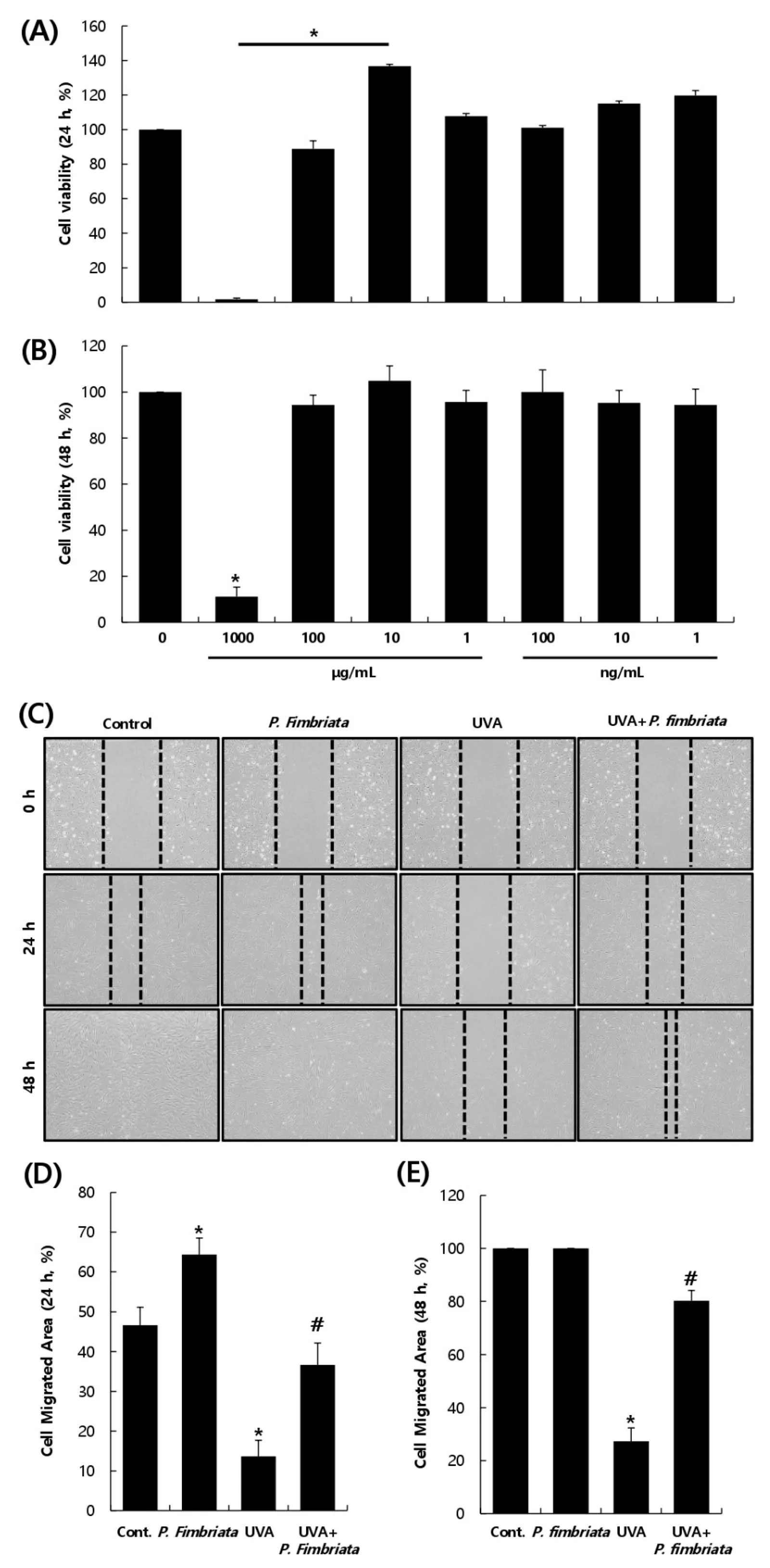
The cytotoxicity and wound-healing effects of *Phycodris fimbriata* extract in normal human dermal fibroblasts (NHDFs). NHDFs were seeded at a density of 3 × 10^3^ cells per well in a 96-well plate and exposed to the specified concentration of *P. fimbriata* extract for 24 h and 48 h. Cell viability was evaluated using the CCK-8 assay; data are presented as the mean ± standard deviation (S.D.) from triplicate measurements for 24 h (**A**) and 48 h (**B**). *P. fimbriata* extract promoted the migration of UVA-irradiated NHDFs. Scratch wound-healing assays were performed over 48 h. Images were captured at 0, 24, and 48 h (magnification: ×100) (**C**–**E**). The experiment was conducted using a *P. fimbriata* extract at a concentration of 10 µg/mL (**C**). Quantitative analysis of the scratch wound healing assay was performed at 24 h (**D**) and 48 h (**E**). The graphs are presented as the mean ± S.D. * denotes significance compared to control. # means compared to UVA-irradiated condition. *, # *p* < 0.05 control group.

**Figure 2:**
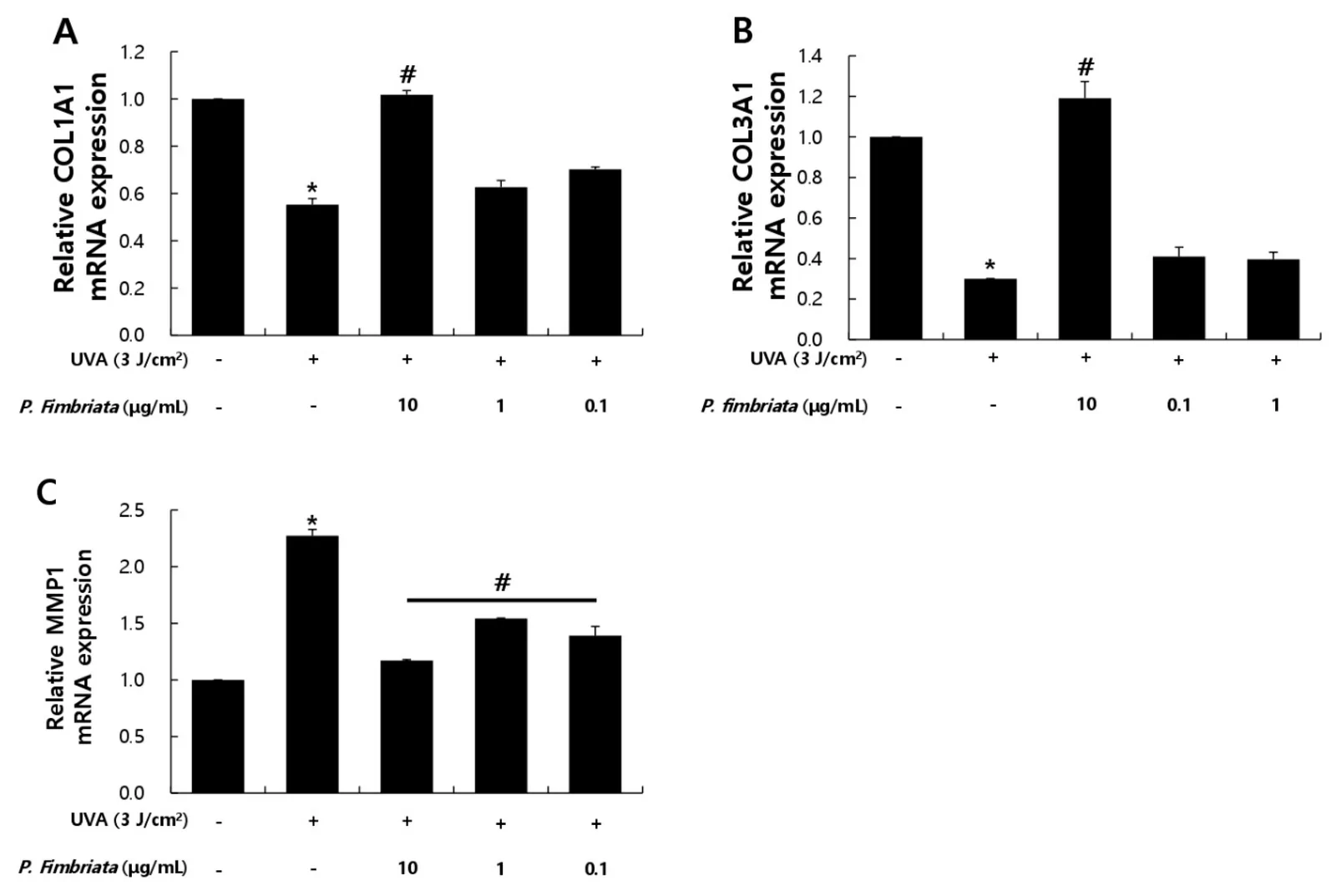
Relative gene expression analysis of *P. fimbriata* extract treatment on UVA-irradiated NHDFs. RT-qPCR analysis was conducted to assess the expression levels of dermal fibroblast markers, including *COL1A1* (**A**), *COL3A1* (**B**), and *MMP1* (**C**). Values are presented as the mean ± S.D. of three independent experiments. * denotes a comparison with the control group; # indicates a comparison with the UVA-irradiated condition. *, # *p* < 0.05. + means treated, - means non-treated.

**Figure 3:**
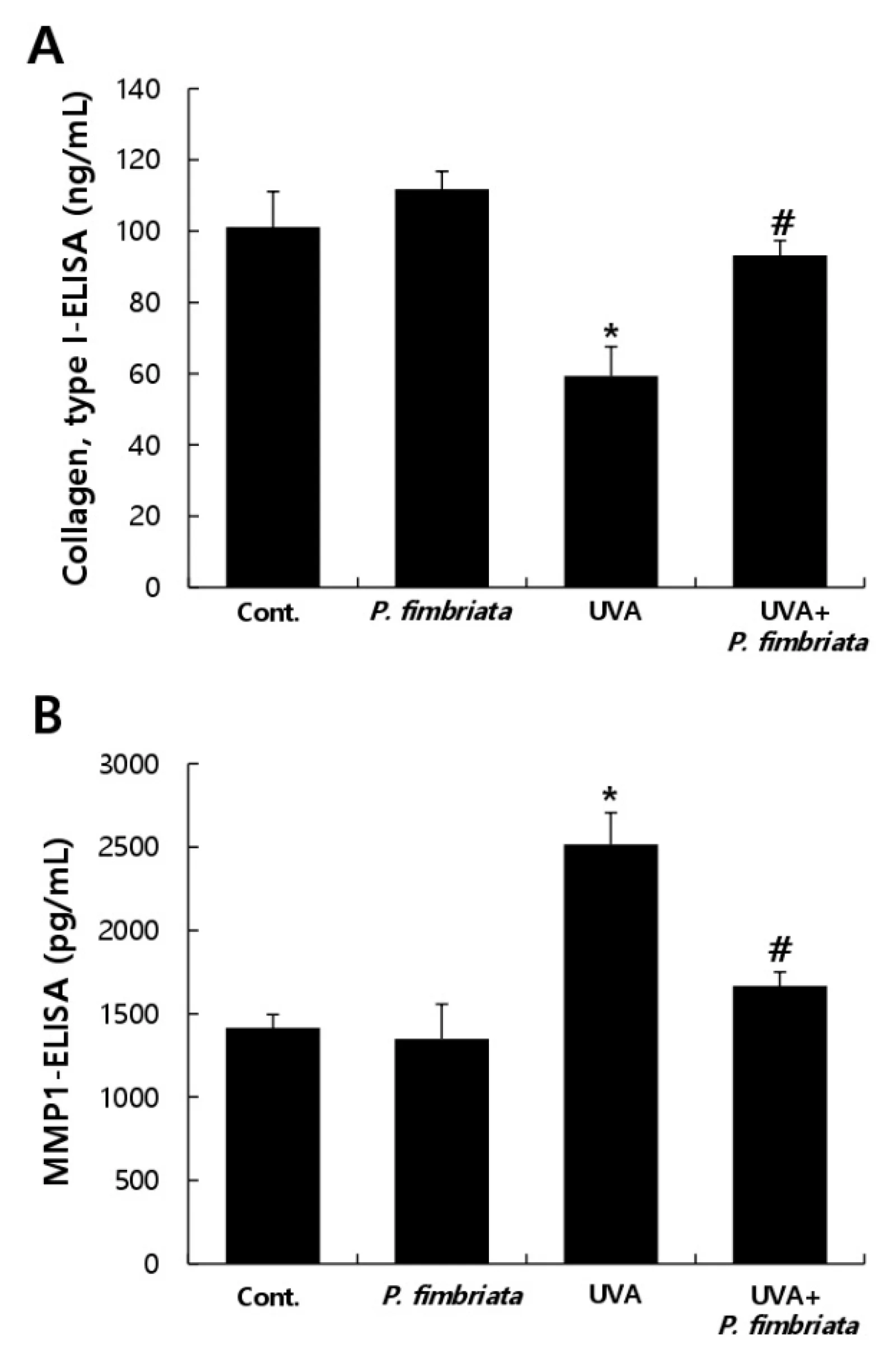
Effects of *P. fimbriata* extract on type I collagen and MMP1 protein synthesis in NHDFs. NHDFs were seeded in a 60 mm tissue culture dish and treated with UVA and *P. fimbriata* extract for 48 h. The experiment was conducted using a *P. fimbriata* extract at a concentration of 10 µg/mL. Conditioned medium was collected and analyzed for type I collagen (**A**), and MMP1 (**B**) ELISA. The data represent the mean ± S.D. of three independent experiments. * denotes a comparison with the control group, # indicates a comparison with the UVA-irradiated condition. *, # *p* < 0.05.

**Figure 4:**
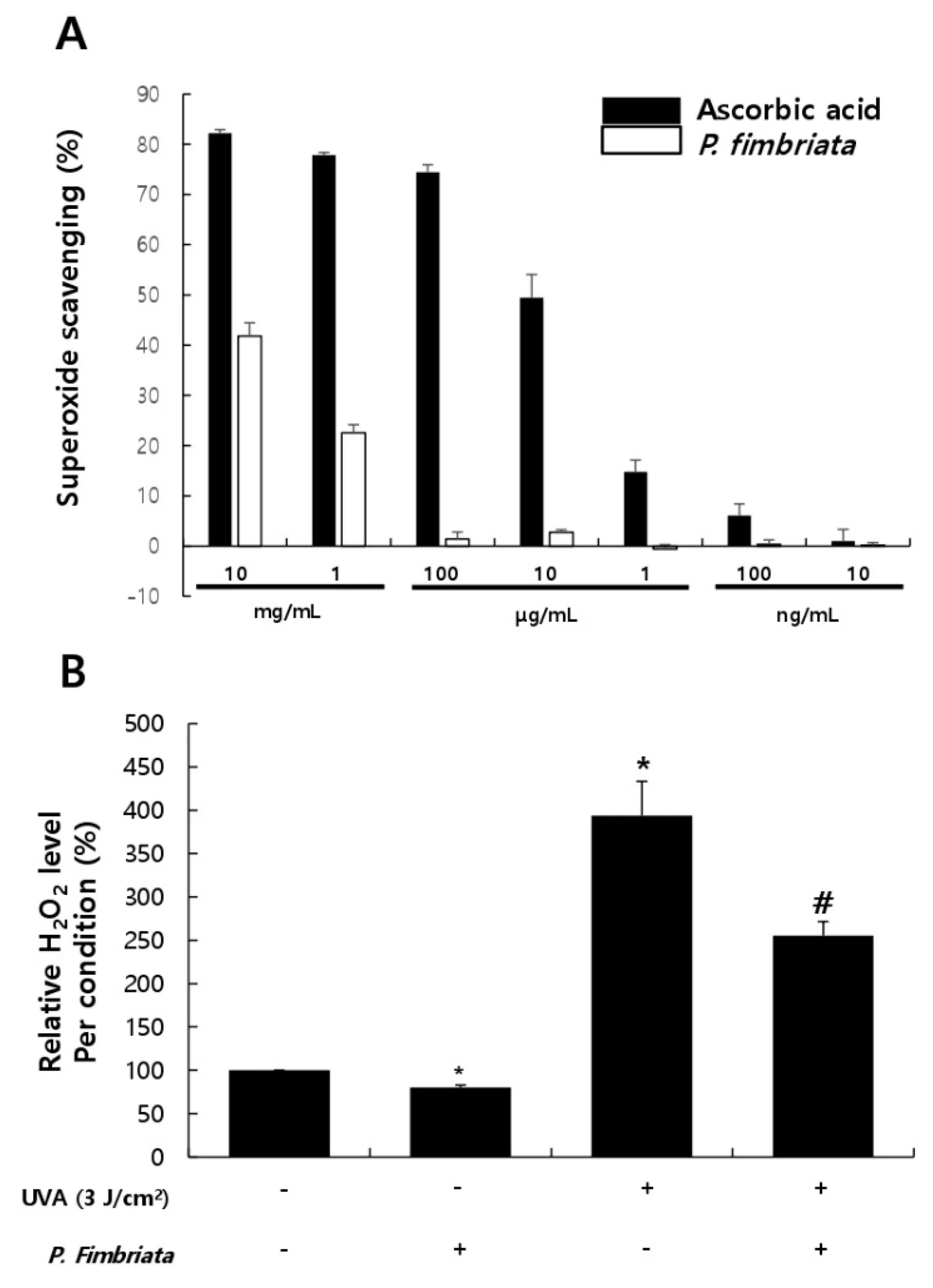
Free radical-scavenging effects of *P. fimbriata* extract. The anti-oxidative effects of *P. fimbriata* extract were evaluated using the DPPH assay at the indicated concentrations (**A**). *P. fimbriata* extract (10 μg/mL) inhibited H_2_O_2_ generation in UVA-irradiated NHDFs (**B**). Graphs depict mean ± S.D. of three independent experiments. * denotes a comparison with the control group, # indicates a comparison with the UVA-irradiated condition. *, # *p* < 0.05. + means treated, - means non-treated.

**Figure 5:**
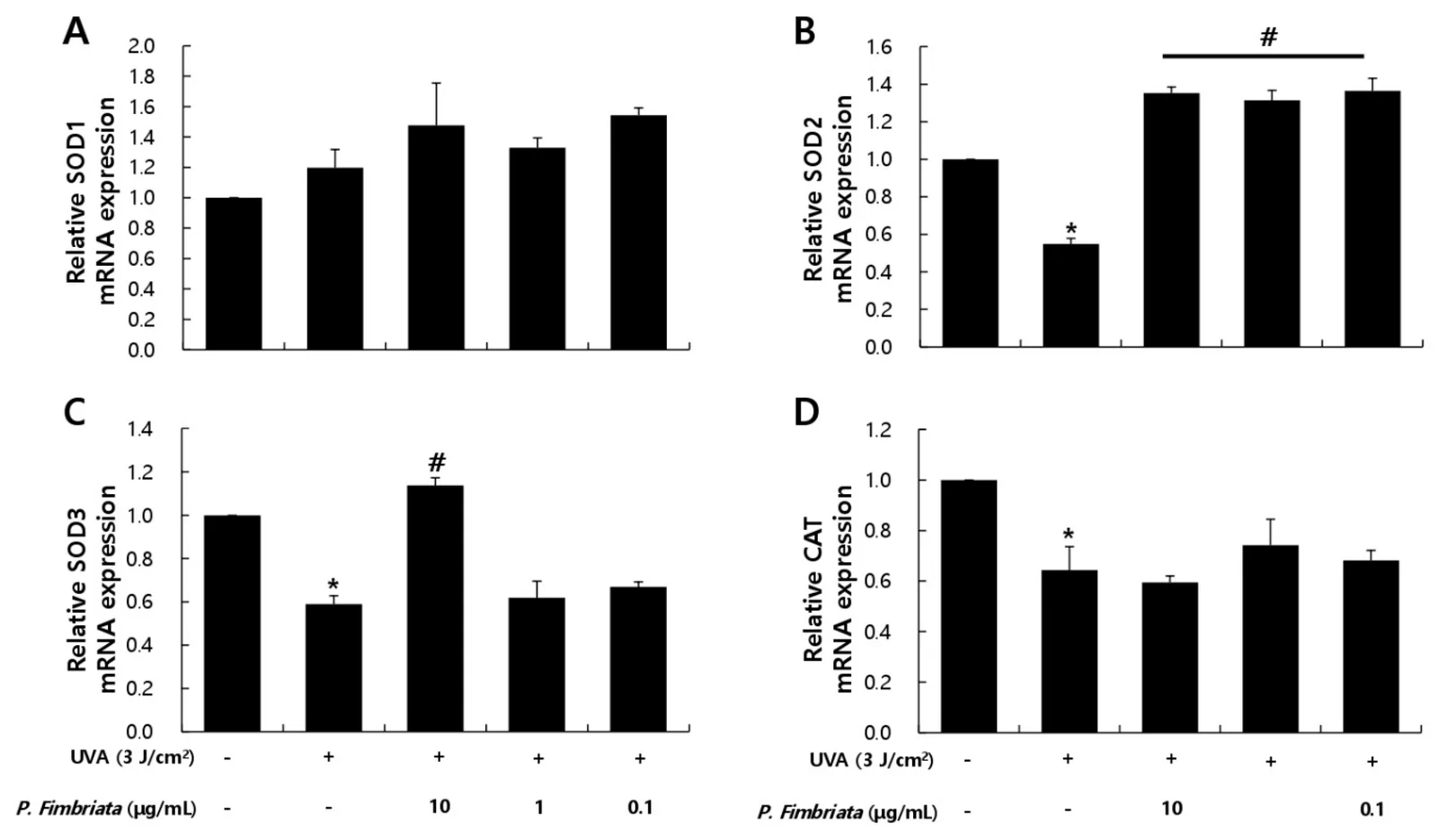
Intracellular antioxidant enzyme expression by *P. fimbriata* extract treatment on UVA-irradiated NHDFs. RT-qPCR analysis was conducted to assess the expression levels of representative antioxidative enzyme markers, including *SOD1* (**A**), *SOD2* (**B**), *SOD3* (**C**), and *CAT* (**D**). The graphs are presented as the mean ± S.D. of three independent experiments. * denotes a comparison with the control group, # indicates a comparison with the UVA-irradiated condition. *, # *p* < 0.05.

**Figure 6:**
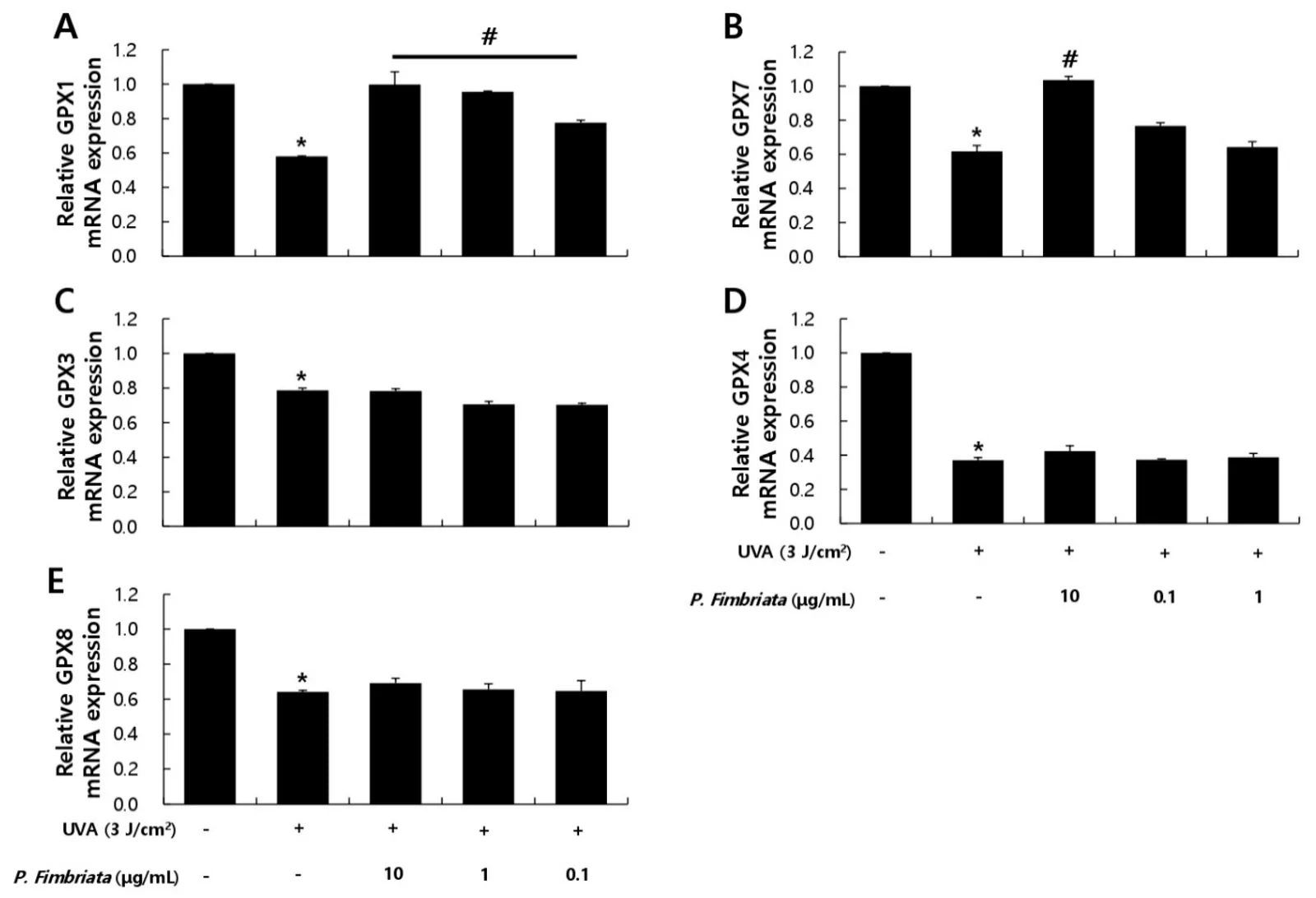
Glutathione peroxidase (*GPX*s) family gene expressions by *P. fimbriata* extract treatment in UVA-irradiated NHDFs. RT-qPCR analysis of the representative antioxidative enzyme markers, *GPX1* (**A**), *GPX7* (**B**), *GPX3* (**C**), *GPX4* (**D**) and *GPX8* (**E**). The graphs are shown as the mean ± S.D. of three independent experiments. * denotes a comparison with the control group, # indicates a comparison with the UVA-irradiated condition. *, # *p* < 0.05. + means treated, - means non-treated.

**Figure 7:**
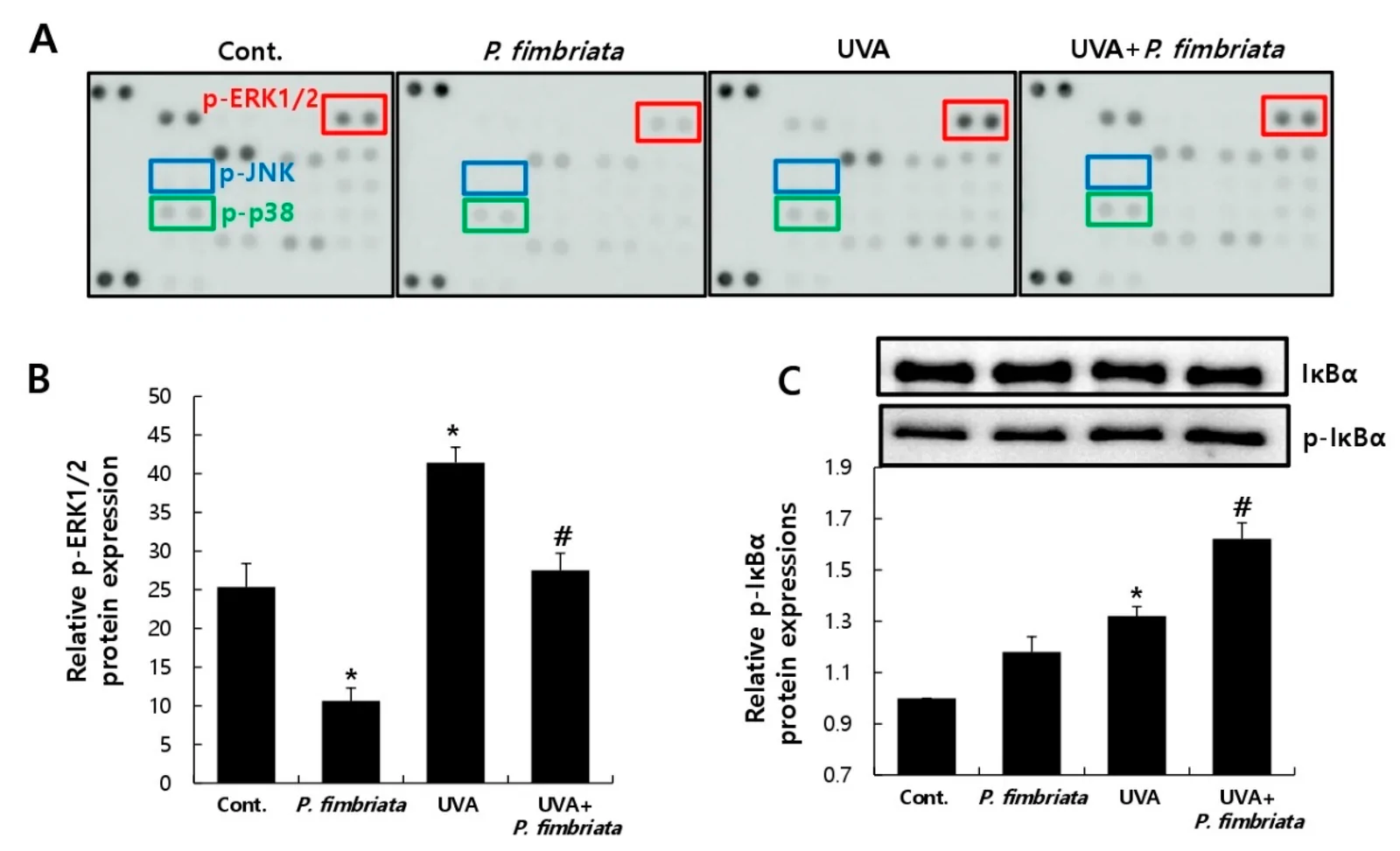
Phosphorylation analysis of intracellular signaling. After collecting whole-cell lysates, lysates were applied to the human phospho-kinase array (**A**,**B**) and Western blotting (**C**). The density ofrelative p-ERK1/2 (**B**) and p-IκBα (**C**) protein expressions. The graphs are shown as the mean ± S.D. of three independent experiments. The experiment was conducted using a *P. fimbriata* extract at a concentration of 10 µg/mL. * denotes a comparison with the control group, # indicates a comparison with the UVA-irradiated condition. *, # *p* < 0.05.

**Figure 8:**
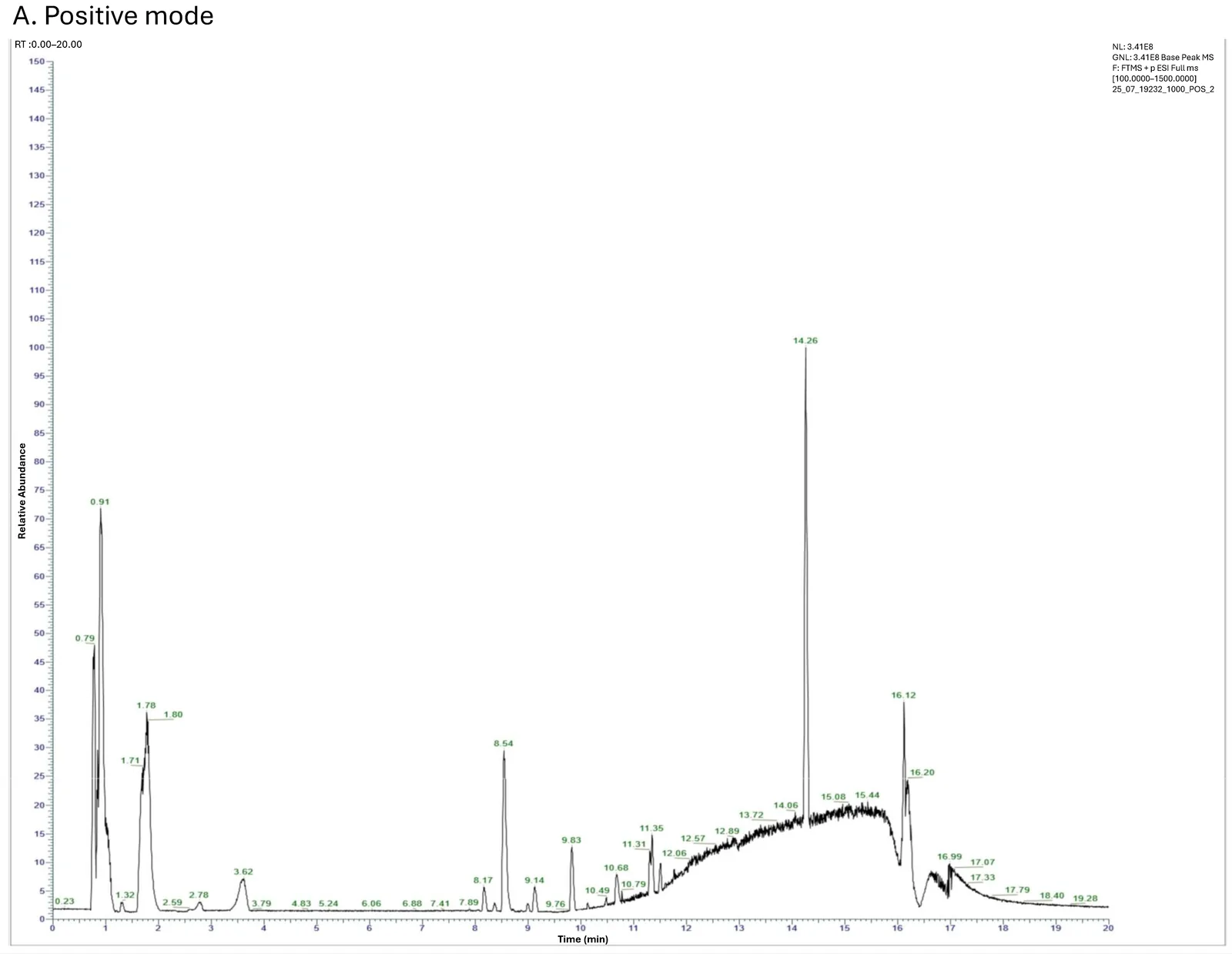

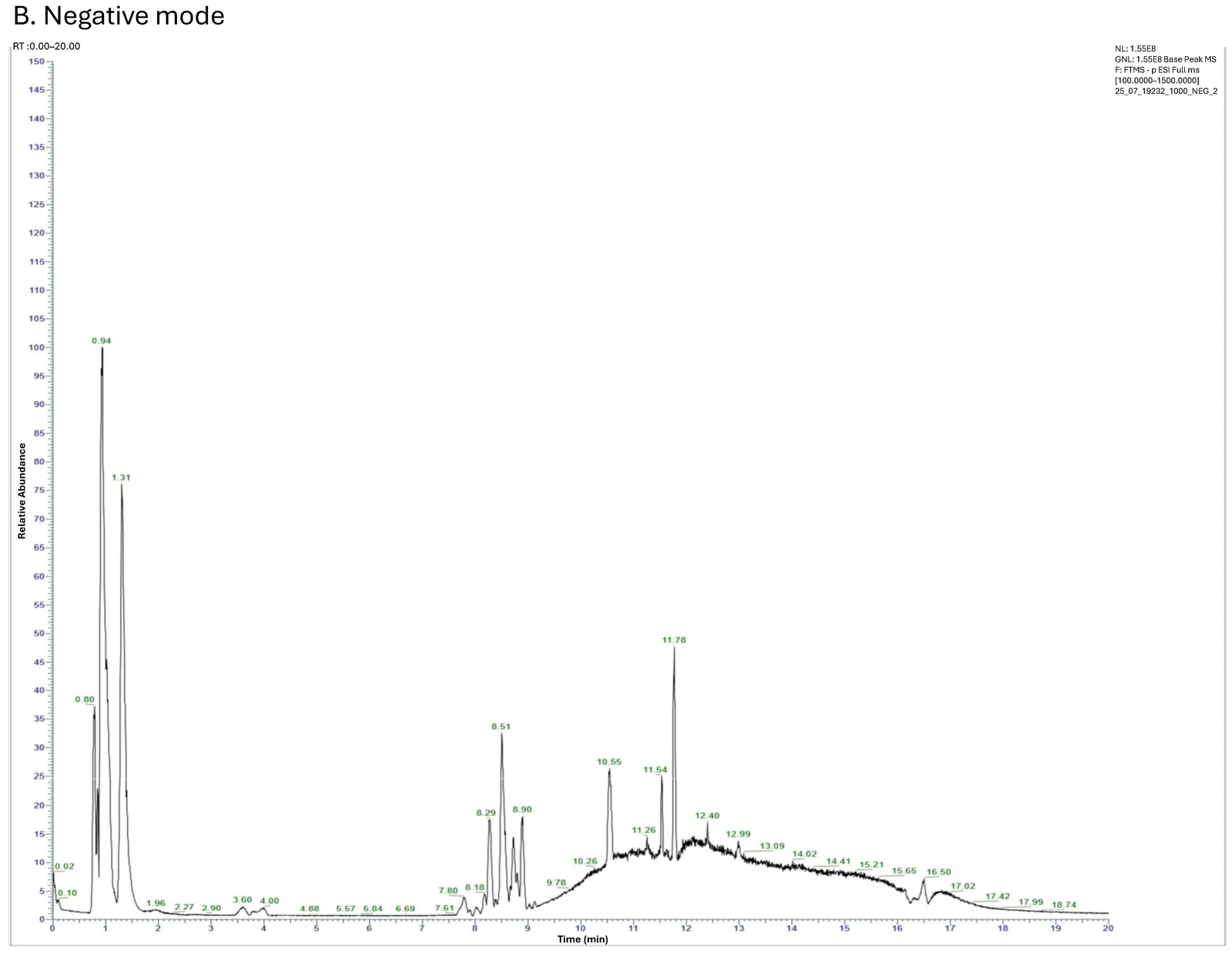
HR-LC/MS Chromatogram of *P. fimbriata* extract. Chromatographic profiles were obtained in both positive (**A**) and negative (**B**) ionization modes to identify unknown components present in the *P. fimbriata* extract.

**Table 1:** Gene Name and Assay ID Number in Real-time RT-PCR Analysis.

Symbol	Gene Name	Assay ID
COL1A1	Collagen, type I, alpha 1	Hs00164004_m1
COL3A1	Collagen, type III, alpha 1	Hs00943809_m1
MMP1	Matrix metallopeptidase 1 (interstitial collagenase)	Hs00899658_m1
SOD1	Superoxide dismutase 1, soluble	Hs00533490_m1
SOD2	Superoxide dismutase 2, mitochondrial	Hs00167309_m1
SOD3	Superoxide dismutase 3, extracellular	Hs04973910_s1
CAT	Catalase	Hs00156308_m1
GPX1	Glutathione peroxidase 1	Hs00829989_gH
GPX2	Glutathione peroxidase 2	Hs01591589_m1
GPX3	Glutathione peroxidase 3	Hs01078668_m1
GPX4	Glutathione peroxidase 4	Hs00989766_g1
GPX5	Glutathione peroxidase 5	Hs00559733_m1
GPX6	Glutathione peroxidase 6	Hs00699698_m1
GPX7	Glutathione peroxidase 7	Hs04194449_s1
GPX8	Glutathione peroxidase 8	Hs00380670_m1
GAPDH	Glyceraldehyde-3-phosphate dehydrogenase	43333764F

**Table 2:** Predicted compounds in the HR-LC/MS library.

Mode	No.	Retention Time (min)	Predicted Compound	CAS No.	Area	Molecular Weight	BM ^c^
Positive ^a^	1	0.857	Choline	67-48-1	490 509 290	103.09966	83.4
	2	0.896	Betaine	107-43-7	251 335 909	117.07895	96.8
	3	0.912	Proline	147-85-3	1 557 356 568	115.06328	100
	4	0.931	4-Aminobenzoic acid	150-13-0	266 595 362	137.04764	64.6
	5	2.790	Phenylacetylene	536-74-3	2 729 747	102.04681	-
	6	8.374	Pyriferine B	-	76 673 555	338.14769	-
	7	9.003	WS79089B	157110-24-2	47 861 03	506.1212	-
	8	9.135	PC (16:0/0:0)	17364-16-8	174 338 170	495.33211	91.4
	9	11.315	Lyso-PAF-C16	52691-62-0	148 103 701	481.35295	100
Negative ^b^	1	1.313	2-Methylcitrate	6061-96-7	815 942 630	206.04214	90.3
	2	3.998	4-Hydroxybenzoic acid	99-96-7	53 481 411	138.03149	99.9
	3	7.796	3-Methoxyphenol sulfate	150-19-6	57 125 113	204.00883	83.3
	4	10.551	N-methyltrosyl-N-methyrosyl-leucyl-alanine	-	275 624 509	556.29048	-

^a^ Positive; *m*/*z* = Molecular weight + 1, [M + H]. ^b^ Negative; *m*/*z* = Molecular weight − 1, [M − H]. ^c^ BM (Best-match score): Predicted compounds in the HR-LC/MS library spectrum in search/data set (mzCloud. mzVault Library) (Results generated from libraries with a BM below 80.0 are considered unreliable.). Analytic condition − Injection volume: 5 μL − Ionization mode: H-ESI − Mass range (*m*/*z*): 100~1500.

## Data Availability

Data is provided within the manuscript.
